# Multiple Ion Scaffold-Based Delivery Platform for Potential Application in Early Stages of Bone Regeneration

**DOI:** 10.3390/ma14247676

**Published:** 2021-12-13

**Authors:** Èlia Bosch-Rué, Leire Díez-Tercero, Raquel Rodriguez-Gonzalez, Román A. Pérez

**Affiliations:** Bioengineering Institute of Technology (BIT), Universitat Internacional de Catalunya (UIC), Sant Cugat del Vallès, 08195 Barcelona, Spain; ebosch@uic.es (È.B.-R.); ldiezter@uic.es (L.D.-T.); rrodriguezg@uic.es (R.R.-G.)

**Keywords:** copper, cobalt, calcium, ion release, bone regeneration, angiogenesis, osteogenesis, antimicrobial properties

## Abstract

Bone has the intrinsic capacity to regenerate itself, as long as the damage is small, through the sequential stimulation of specific phases, such as angiogenesis followed by osteogenesis. However, when the damage is extensive it is unable to regenerate and bone tissue engineering is used as an alternative. In this study, we developed a platform to allow the triple ion delivery with sequential delivery capacity to potentially stimulate antibacterial, angiogenic and osteogenic processes. The scaffold-based platform consisted of alginate/hydroxyapatite (HA) microparticles embedded in alginate fibers. Firstly, microparticles were developed using different ratios of alginate:HA using the spraying method, resulting in a high reproducibility of the technique. Microparticle size between 100–300 µm and ratio 1:40 resulted in a more spherical morphology and were selected for their incorporation into alginate fiber. Different amounts of copper and cobalt were added with the microparticles and alginate fiber, respectively, were used as model ions which could eventually modulate and mimic antimicrobial and angiogenic processes. Moreover, calcium ion was also incorporated in both, in order to provide the system with potential osteogenic properties together with HA. The multiple delivery of copper, cobalt and calcium released were in the therapeutic range as measured by induced coupled plasma (ICP), providing a promising delivery strategy for tissue engineering.

## 1. Introduction

Bone tissue has the ability of self-regeneration in response to small size damage, although it is unable to regenerate tissue damage with a size higher than 2.5 cm [1]. In this last case, clinical intervention using autologous or allogeneic bone grafts have been the gold standard treatment for a long time, although they possess some disadvantages such as availability of the graft, possible immune response and possible risk of disease transmission [2,3]. Therefore, the development of synthetic grafts for bone tissue engineering (BTE) emerged as an alternative source for bone regeneration. First attempts in BTE resulted in the use of biological inert materials to just fill the bone defect volume [4]. Over the years, those scaffolds have evolved to meet some characteristics, such as improving their ability to induce osteogenic differentiation through the release of stimulatory molecules, and preferably with a customized shape in order to fill different bone defects, amongst others [5,6]. Considering that native bone regeneration involves multiple steps (inflammation, angiogenesis, chemotaxis and osteogenesis) and the release of multiple signaling molecules [7], scaffolds developed as multi drug delivery systems that mimic these phases have been considered more effective for the regeneration of large bone damage [8]. Among the stimulatory molecules incorporated within bone scaffolds, growth factors or antibiotics (to avoid infection upon scaffold implantation) have been extensively used. For instance, Hsu et al., developed poly(D,L)-lactide-*co*-glycolide (PLGA) core–shell nanofibers where vancomycin and ceftazidime, two antibiotics, were encapsulated in the shell, and the osteogenic growth factor bone morphogenetic protein 2 (BMP-2) in the core [9], with the aim to avoid infection upon implantation and stimulate osteogenesis. Results demonstrated dual delivery of these molecules, avoiding bacteria growth and enhancing osteogenic differentiation with enhanced alkaline phosphatase (ALP) activity. As another example, in order to stimulate angiogenesis followed by osteogenesis, Kanczler et al., developed alginate fibers embedded in polylactic acid (PLA) matrix containing vascular endothelial growth factor (VEGF) and BMP-2, respectively [10]. VEGF was first released from day 7, and thereafter, BMP-2 was released up to 28 days, demonstrating an increased bone formation with an in vivo femoral bone mouse defect. Despite their potential, antibiotics can induce antimicrobial resistance, considered one of the biggest threats to global health according to World Health Organization [11], while growth factors have delicate handling properties and short half-life. As an alternative, ions have been considered as attractive option as they have been described to induce therapeutic effects, with the ability to regulate cellular functions similar to growth factors presenting higher stability [7]. Examples of therapeutic roles are anti-bacterial, described with silver, copper, zinc, gallium [12]; anti-inflammatory, reported with magnesium or zinc [13,14]; angiogenic, demonstrated with copper, cobalt and silicon [15]; or osteogenic, described with silicon, zinc or strontium [7,15]. However, it is of special importance the amount of ion used as they can induce cytotoxic effects when surpassing the therapeutic window [16].

Ions can be incorporated within the structure of biomaterials trough different approaches. For instance, ceramics can be doped with ions to allow its incorporation within their chemical structure using high temperatures [16,17]. Nevertheless, their release depends entirely on the crystallography of the designed ceramic, together with a limited ability to encapsulate other molecules within the structure due to the high temperatures required. Another less time-consuming method is based on the introduction of ions in the crosslinking solution of polymers, which can be performed at room temperature, allowing at the same time the incorporation of molecules sensible to temperature such as growth factors. For instance, a previous study reported the development of alginate–collagen core–shell structures where cobalt ion was incorporated in the outer layer through alginate crosslinking solution and BMP in the inner core, enhancing in vivo angiogenesis and bone formation [18]. The crosslinking of polymers such as alginate has as well allowed the allocation of hydroxyapatite (HA), which can allow inducing to a higher extent specific regeneration processes, such as osteogenesis [19]. Furthermore, the combination of both materials in a spherical morphology has been previously shown to allow more homogenous delivery based on the spherical morphology [20,21,22]. Moreover, the loading amount of drug that can be incorporated can be controlled by increasing or reducing their size. Nonetheless, taking into account that microspherical carriers can deliver one or more than one molecule, but all of them simultaneously; in order to achieve a sequential release they can be embedded in another matrix, to allow multiple delivery of factors/ions. As an additional advantage, this opens the possibility to extrude them in a 3D printer, with the benefit of custom-shape scaffolds for different bone defects [23].

The aim of this study is to develop a scaffold-based platform to allow multiple ion delivery and find different release patterns for future applications in bone regeneration. To this purpose, the development of hydroxyapatite (HA)–alginate-based microparticles and their incorporation within alginate fiber structure was first established. Then, copper and cobalt ions were introduced in the microparticles and fiber, respectively. To the best of our knowledge, the combination of these ions has not been previously studied in HA and alginate-based scaffolds. Additionally, our null hypothesis is that the release of therapeutic ions from the microparticles and fiber can be modulated by changing their concentrations in the crosslinking solutions.

## 2. Materials and Methods

### 2.1. Study Design

**Figure 1:**
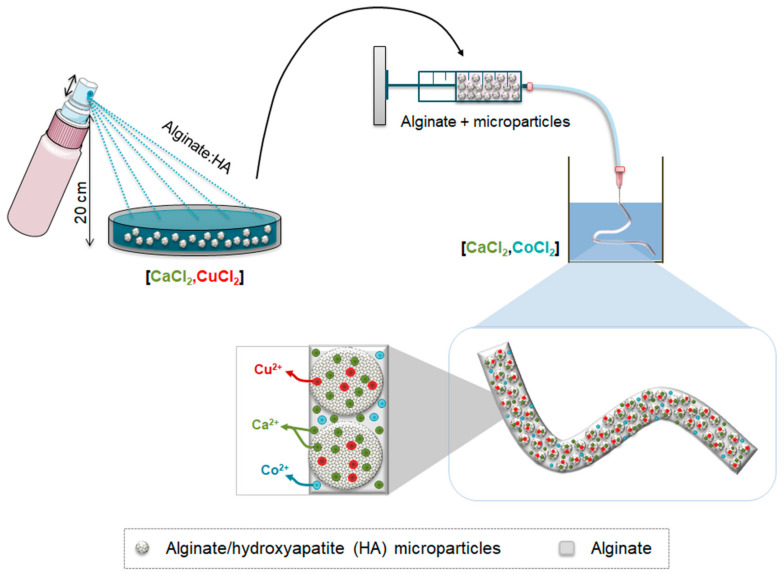
Schematic representation of the development of alginate/hydroxyapatite (HA) microparticles and their subsequent encapsulation in alginate fibers. First, a blend of alginate/HA was loaded in a fingertip spray and the mixture was sprayed in a crosslinking solution containing different concentrations of calcium and copper. After gelation, microparticles were loaded in a syringe containing alginate solution. With the help of an injection pump, the alginate containing alginate/HA microparticles were extruded in a crosslinking solution containing different concentrations of calcium and cobalt.

The intention of the ion delivery platform was to allow a fast burst release of a model ion that could eventually have antimicrobial activity, followed by a slightly sustained release of a model ion that could stimulate angiogenesis, combined with a release of Ca^2+^ which could eventually aid the angiogenesis as well as the osteogenesis. As the proposed ions are being used as model ions, this system could eventually be tuned with different ions depending on the phases of tissue regeneration that need to be triggered. Hence, in this study a triple ion delivery system is developed, consisting of an alginate fiber loaded with alginate/hydroxyapatite (HA) microparticles. For this purpose, copper (Cu^2+^) was selected as the antimicrobial model ion, whereas cobalt (Co^2+^) was selected as the angiogenic model ion. Initially, we first optimized the development of alginate/HA microparticles and alginate monofibers, separately, using a calcium (Ca^2+^) based crosslinking solution. Then, we optimized the amounts of copper (Cu^2+^) to be incorporated in the crosslinking solution to allow its incorporation within the microparticles as well as the amounts of cobalt (Co^2+^) to be incorporated in the crosslinking solution to allow its incorporation in the fibers (Figure 1).

### 2.2. Cross-Linked Alginate/HA Microparticle Development

As a first step, alginate microparticles containing hydroxyapatite (HA) were optimized. For this purpose, 0.5% (%wt) alginate was used and different amounts of hydroxyapatite (particle size 2.5 µm, Sigma-Aldrich, Saint Louis, MO, USA) were added, with ratios %*w*/*w* alginate:HA of 1:0, 1:4, 1:40, and 1:50 dissolved with mili-Q H_2_O. The aim was to incorporate the maximum amount of HA to increase the osteogenic properties of these microparticles. The resultant mixture was introduced in a fingertip spray bottle (DDBiolab). Microparticles were generated by spraying the sample into 150 mM CaCl_2_ contained within a non-adhesive circular container at 20 cm of distance. Once the sprayed solution made contact with the surface of the CaCl_2_ solution, instant crosslinking occurred. After 5 min of incubation, microparticles were sieved with different pore size meshes (DDBiolab) between 50 and 100 µm and between 100 and 300 µm with the aid of mili-Q H_2_O. Fractioned microparticles were kept in mili-Q H_2_O and used thereafter.

The size distribution of sieved microparticles was assessed by Laser diffraction using the Universal Liquid Module of LS13320 (Beckman). Initially, the size distribution of the different ratios tested (1:0, 1:4 and 1:40) was evaluated, with the exception of 1:50, as it could not be sprayed and hence this composition was discarded as a viable ratio for microparticle preparation. Then, the particle size distribution of the sieved microparticles was analyzed in order to confirm that the size of the microparticles were in the sieved range. We sprayed three times the different ratios to obtain three triplicates per condition. The microparticles with the highest amount of HA incorporated and higher size were considered optimal, due to their osteogenic potential properties and higher loading capacity, respectively, and were further used for the ion delivery system.

For the incorporation of Cu^2+^ within alginate/HA microparticles, the intention was to use a CuCl_2_ crosslinking solution of 150 mM as it previously demonstrated to be enough for alginate gelation. However, as higher concentrations of Cu^2+^ can be cytotoxic for cells, we combined it with Ca^2+^ which has been previously demonstrated to not be harmful at these levels [18]. The different combinations of Ca^2+^ and Cu^2+^ that were incorporated in the crosslinking solutions are shown in Supplementary Table S1, following the same procedure explained in this section.

### 2.3. Cross-Linked Alginate Fiber Development

As a second step, alginate fiber development was optimized. For this purpose, several parameters were addressed, which included alginate concentration, syringe needle and injection speed. First, sodium alginate (PanReac Applichem) was dissolved with mili-Q H_2_O and loaded into 5 mL syringes (Becton Dickinson). The alginate concentrations used were those that allowed fiber shape structure when extruded and, at the same time, that allowed their manipulation without breaking. For this purpose, the concentrations tested were 0.5% and 1.5% (%wt). Alginate solutions were extruded in a 150 mM CaCl_2_ crosslinking solution through different sizes of syringe needles. More specifically, the sizes tested were 20 G (⌀_in_: 0.65 mm), 25 G (⌀_in_: 0.32 mm), 26 G (⌀_in_: 0.26 mm) and 27 G (⌀_in_: 0.22 mm) (Becton Dickinson). Different constant injection speeds (10, 50, 100 and 150 mL/h) were studied with the aid of a syringe pump (KD Scientific). After 5 min of incubation in the CaCl_2_ solution, fibers were washed three times with mili-Q H_2_O in order to remove the excess of ions. Fibers were done in triplicates for each condition and imaged with the help of a Stereo Discovery v8 microscope coupled to an Axiocam ERc 5s camera (Zeiss). Ten measurements of the diameter per fiber were made to assess how the concentration of alginate, the syringe needle size and the injection speed affected the size of the fiber.

In order to incorporate Co^2+^ within alginate fiber, different combinations of Ca^2+^ and Co^2+^ were incorporated in the crosslinking solution, shown in Supplementary Table S2, following the procedure described in this section. Similar to Cu^2+^, Co^2+^ ions were also combined with Ca^2+^ to avoid the release of higher concentrations with the potential to be cytotoxic.

### 2.4. Development of Triple Ion Delivery System

As previously mentioned, the aim was to incorporate the maximum amount of microparticles within the alginate fiber to provide it with osteogenic properties. For this purpose, different amounts of microparticles (suspended with the minimum mili-Q H_2_O solution that allowed their pipetting) were mixed with alginate fiber solution by volume (%*v*/*v*). The amounts incorporated ranged from 10 to 60% (%*v*/*v*). In all the cases, the final concentration of alginate fiber was 1.5% (%*w*/*v*). For further experiments, we selected the amount of microparticles that allowed the continuous extrusion of the fiber. The size of fibers with the maximum amount of microparticles incorporated was measured with the help of a Discovery v8 microscope coupled to an Axiocam ERc 5s camera (Zeiss).

### 2.5. Release of Copper, Cobalt and Calcium

The ion release of different combinations of Ca^2+^ and Cu^2+^ from alginate/HA microparticles embedded within alginate fibers cross-linked with different combinations of Ca^2+^ and Co^2+^ were studied. Different combinations of ions are shown in Table 1.

The ion release study was performed under sterile conditions. Briefly, alginate and hydroxyapatite powders were sterilized under three rounds of 15 min each one with UV. After sterilization, they were resuspended with mili-Q H_2_O previously filtered with 0.2 µm filters. Crosslinking solutions of CaCl_2_, CuCl_2_ and CoCl_2_ were also filtered with 0.2 µm filters for their sterilization. The fingertip spray bottle, the mili-Q H_2_O container and meshes were sterilized with gas plasma (Johnson & Johnson). The non-adhesive circular container was autoclaved.

For each condition, triplicates of 0.15 mL of alginate fibers containing microparticles were extruded and after 5 min of incubation, they were soaked 3 times with cell culture media to remove the excess of ions. Subsequently, each fiber was incubated with 1 mL of endothelial growth medium (EGM-2) (Lonza) at 37 °C for a total duration of 7 days to simulate physiological conditions. Supernatants were collected at 1, 3, 5 and 7 days. For their analysis, each mL of supernatant was diluted with 9 mL of 2% HNO_3_ (dilution 1:10), and kept at 4 °C until analysis. The release of Ca^2+^ ions were analyzed with inductively coupled plasma–optical emission spectrometry (ICP-OES) and the release of Cu^2+^ and Co^2+^ were analyzed with inductively coupled plasma–mass spectrometry (ICP-MS). Cell culture media without fiber incubation was used as blank.

### 2.6. Statistical Analysis

Statistical analysis was performed using IBM SPSS statistics 21.0 software (IBM Corp., Armonk, NY, USA). Kruskal–Wallis and Mann–Whitney U non-parametric tests were used to compare microparticle mean size from different microparticle compositions (triplicate experiment), the size of alginate fiber depending on the injection speed (n = 12) and the size of pristine alginate fibers and fibers with the maximum microparticles incorporated (n = 3). Statistically significant differences were considered with *p* < 0.05.

## 3. Results

### 3.1. Microparticle Size Distribution

For microparticle development, HA amount was increased to determine the maximum content of HA that could be sprayed. Alginate:HA ratios up to 1:40 could be easily sprayed, while higher ratios (1:50) partially obstructed the fingertip spray. To assess if this method was reproducible to develop microparticles, triplicates of the size distribution were analyzed with an alginate:HA ratio of 1:0, 1:4 and 1:40.

**Figure 2:**
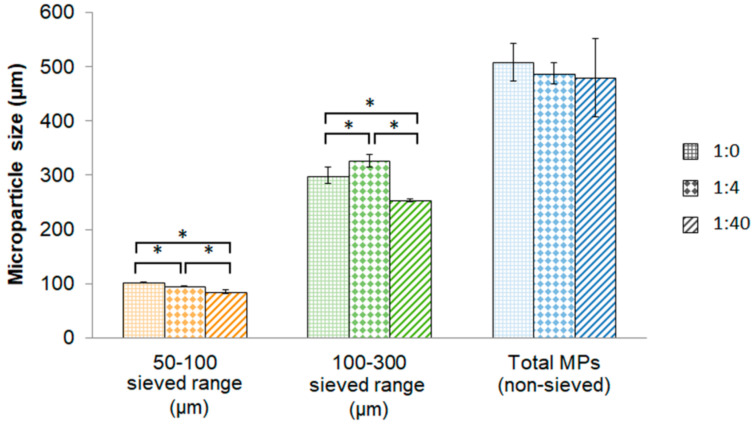
Mean size of microparticles. Graphical representation of mean size of sieved (50–100 and 100–300 µm) and non-sieved microparticles for different alginate/HA ratios (1:0; 1:4 and 1:40). Abbreviations: MPs = microparticles. * Statistically significant differences (n = 3) (*p* < 0.05).

Un-sieved microparticles of ratios 1:0, 1:4 and 1:40 did not present differences in the mean size (Figure 2). However, sieved microparticles presented significant differences between different microparticles compositions, presenting the smaller mean size with the maximum amount of HA incorporated. More in detail, the size of the microparticles with the 1:40 ratio were 85.3 ± 2.8 µm with 50–100 µm size range and 253.6 ± 2.0 µm with 100–300 µm size range. Moreover, the coefficient of variation obtained was low in all compositions tested, indicating reproducibility of this technique.

**Figure 3:**
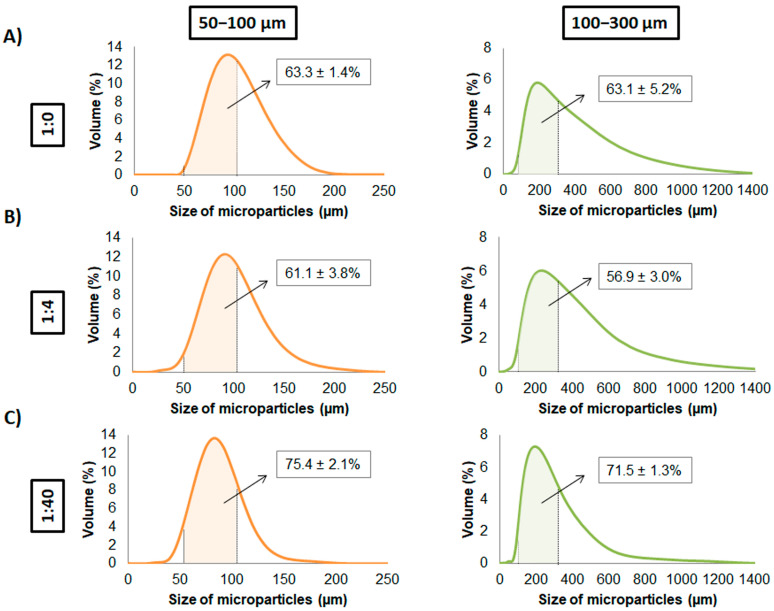
Size distribution of sieved alginate/HA microparticles. Mean size distribution (n = 3) is represented for 50–100 and 100–300 µm microparticles for alginate:HA ratios of (**A**) 1:0, (**B**) 1:4 and (**C**) 1:40. Percentage of microparticles between the size range of 50–100 and 100–300 µm is represented within the respective size distribution graphs.

After sieving the microparticles, their size distribution (Figure 3) and the percentage of microparticles that had a size between 50–100 and 100–300 µm was assessed. Regarding the size distribution, the condition 1:40 presented a more uniform size compared to other conditions either in 50–100 and 100–300 µm. Concerning the percentage of microparticles in each size range, with the ratio 1:0, the respective amounts with 50–100 µm and 100–300 µm were 63.3 ± 1.4% and 63.1 ± 5.2%, respectively (Figure 3A). With the ratio 1:4, the amount obtained were 61.1 ± 3.8% and 56.9 ± 3.0% with 50–100 µm and 100–300 µm sieved microparticles, respectively (Figure 3B). Finally, with the ratio 1:40, an amount of 75.4 ± 2.1% and 71.5 ± 1.3% were obtained with 50–100 µm and 100–300 µm sieved microparticles, respectively (Figure 3C). Therefore, the ratio 1:40 was the one with the highest percentage of microparticles within the size ranges, suggesting homogenous size compared to other conditions.

**Figure 4:**
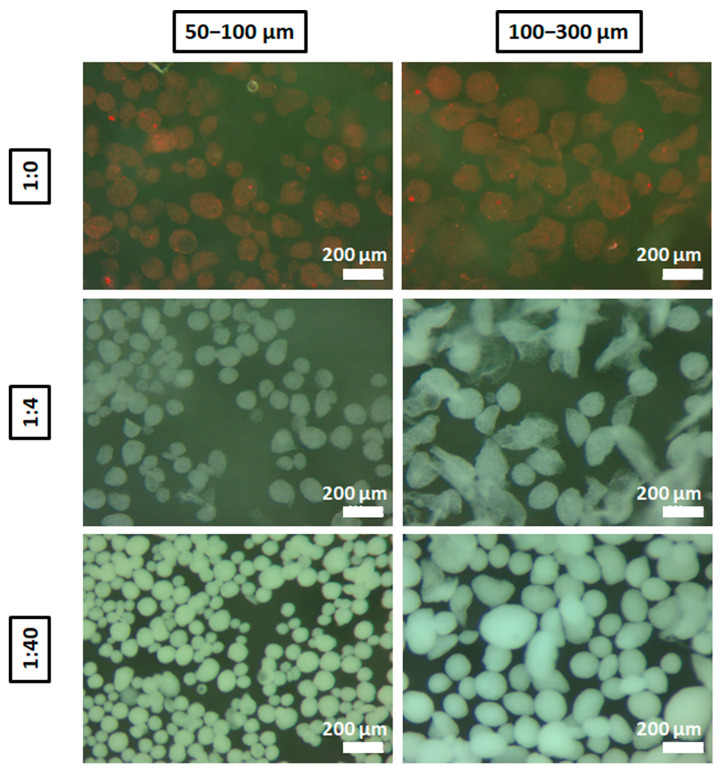
Stereomicroscope images of sieved microparticles. Images of microparticles between 50–100 and 100–300 µm from different alginate:HA ratios: 1:0 (stained with red ink for their visualization), 1:4 and 1:40. Scale bar = 200 µm.

The morphology of the microparticles was then assessed with stereomicroscope showing that, in general, 50–100 µm microparticles presented homogeneity and sphericity in all compositions (Figure 4). It is important to highlight that the ratio 1:40 maintained the morphology more spherical with the size range of 100–300 µm compared with 1:4 and 1:0, and this might explain why 1:40 ratio condition had a more homogenous size after sieving. It is noteworthy that microparticles could be manipulated without jeopardizing their structure.

For further experiment analysis, the alginate:HA ratio of 1:40 was considered, as it allows the maximum hydroxyapatite incorporation. Furthermore, the size range of 100–300 µm was selected to be used for subsequently assays, as this size range would allow a higher ion loading capacity.

### 3.2. Alginate Fiber Size

Taking into account that these microspheres in the range of 100–300 µm are incorporated within the alginate fiber, the size and stability of the fibers had to be determined prior to microspheres incorporation. In this sense, fiber size should eventually be in the range size of 500 µm to adequately allocate the microspheres. Moreover, it should be extruded in short period of time and be reproducible as well as present enough mechanical stability. For this purpose, the effect of different variables (alginate concentration, needle diameter (G) and injection speed) on the final alginate fiber diameter was assessed, in order to choose the most appropriate parameters to encapsulate microparticles within them.

**Figure 5:**
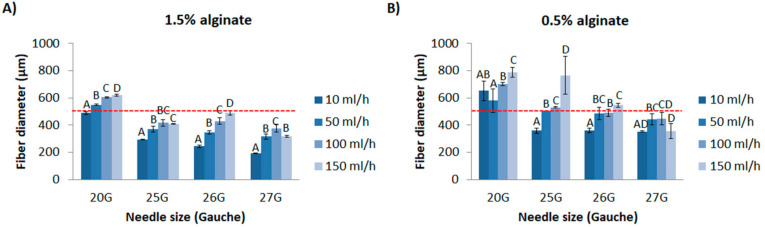
Alginate fiber size. Representation of alginate fiber size depending on the injection speed with (**A**) 1.5% or (**B**) 0.5% of alginate. Statistics is represented with letters when comparisons were made between the different injection speeds for each needle size. Same letters represent no significant differences (*p* > 0.05), whereas different letters represent statistical differences (*p* < 0.05) (n = 12). Red dashed line indicates an appropriate size of alginate fiber diameter to encapsulate alginate/HA microparticles from 100 to 300 µm.

Regarding the injection speed, in general it was observed that its increase resulted in an increase of alginate fiber diameter as represented in Figure 5. The lowest concentration of alginate of 0.5%, in general presented higher diameters compared to 1.5% concentration. Moreover, 0.5% presented higher standard deviation and coefficient of variation in more than half of the conditions compared to 1.5%, suggesting higher homogeneity of fiber size with 1.5% alginate (Figure 5). Moreover, needle G also presented an effect on the final fiber diameter, obtaining the higher sizes with the higher inner diameter of the needle (20G).

As one of the aims was to incorporate microparticles with a size range of 100–300 µm within alginate fibers, the use of 20G needles was preferred since their inner diameter is 650 µm and it would avoid possible obstructions. Additionally, 1.5% alginate concentration presented more fiber size homogeneity and at the same time, it was more consistent allowing handling without breaking compared to 0.5%. Therefore, 1.5% concentration was used for the following experiments. Taking into account that higher speeds lead to higher shear stresses and possible microparticle fracture, injection speeds of 100 and 150 mL/h were considered too high and were therefore discarded. Alternatively, 10 and 50 mL/h present lower and similar flow rate, so it was decided that 50 mL/h injection speed would be less time consuming. Additionally, the fiber size obtained with 50 mL/h is higher than 10 mL/h, specifically 550.5 µm and 488.7 µm, respectively, therefore providing more space to accommodate big size microparticles.

### 3.3. Microparticle Incorporation within Alginate Fibers

**Figure 6:**
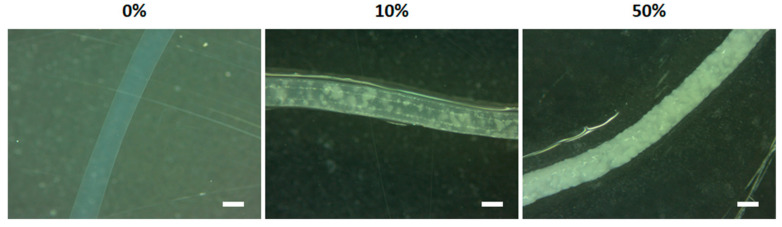
Incorporation of alginate/HA composite microparticles within alginate fibers. Images of pristine alginate fibers (**left**), the minimum (10%) amount of microparticles incorporated (%*v*/*v*) (**middle**) and maximum amount (50%) that could be incorporated avoiding fiber rupture (**right**). Scale bar = 500 μm.

Finally, the maximum amount of microparticles with a size of 100–300 µm (1:40 ratio) was incorporated within the alginate fiber. Increasing concentrations starting at 10% (%*v*/*v*) were used for this purpose. The low filling density with 10% (%*v*/*v*) (Figure 6) was progressively increased by increasing the amount of microparticles with 20, 30, 40 and 50% (%*v*/*v*). The 50% (%*v*/*v*) amount was the maximum that could be incorporated within alginate fiber without rupture and needle obstruction, and also could maintain homogeneous diameter size as shown in Figure 6. The size of alginate fibers containing 50% of 1:40 microparticles was in a similar size range compared to the pristine alginate fiber, presenting only an increase of 40 µm (595.8 ± 25.9 µm). For further experiments, microparticles within fibers with a ratio of 50% (*v*/*v*) were used. It is worth to mention that with this amount of microparticles loaded, they were close to the surface with an almost direct contact to the exterior.

### 3.4. Ion Release

**Figure 7:**
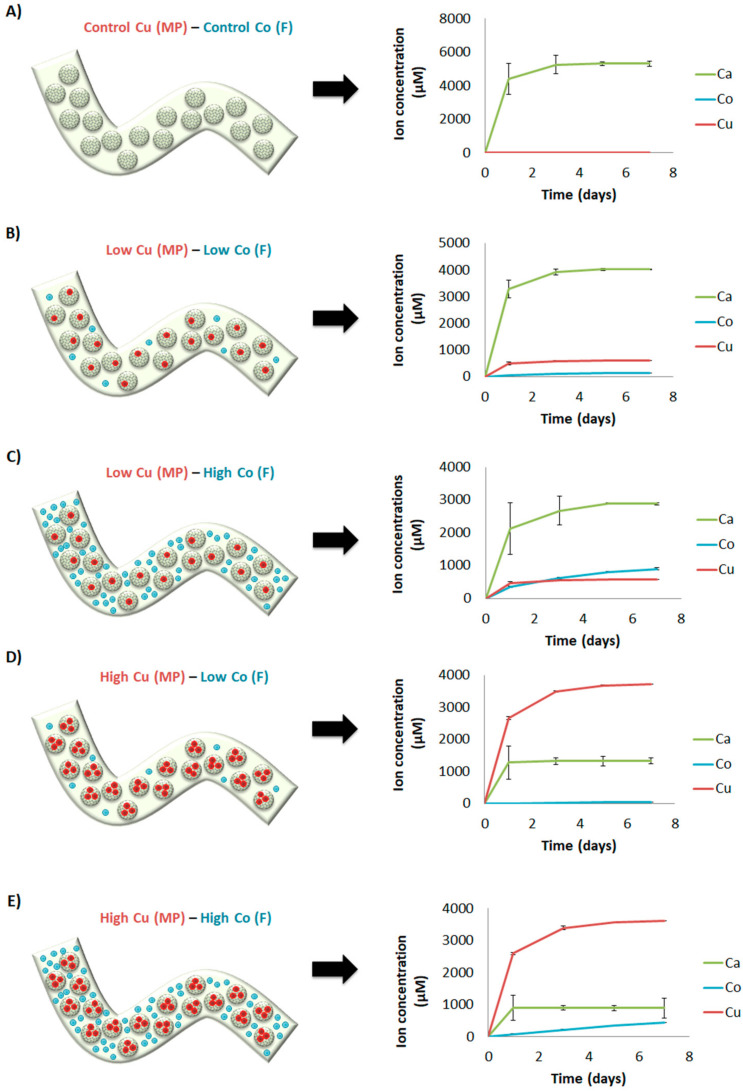
Sustained release of calcium, cobalt and copper ions. Different concentrations of calcium, cobalt and copper were incorporated in the dual drug delivery system using different crosslinking solutions with different ion concentrations (microparticle-fiber): (**A**) Control Cu (MP)—Control Co (F), (**B**) Low Cu (MP)—Low Co (F), (**C**) Low Cu (MP)—High Co (F), (**D**) High Cu (MP)—Low Co (F), (**E**) High Cu (MP)—High Co (F). Release of ions measured up to 7 days. Abbreviations: MP = microparticles; F = fiber.

The crosslinking solutions allowed incorporating the different ions in the delivery platform. Although the exact amount of ions incorporated was unknown, we could measure their release. In general, Ca^2+^ and Cu^2+^ presented a burst release profile, whereas Co^2+^ presented a more sustained release close to zero order kinetics. More in detail, when Co^2+^ and Cu^2+^ were not incorporated in the ion delivery system (control Cu (MP)—control Co (F)), Ca^2+^ showed the maximum concentration delivered (Figure 7A). With the low concentration of Cu^2+^ and Co^2+^ in the microparticles and within the fiber (low Cu (MP)-low Co (F)), respectively, the release of calcium was reduced (Figure 7B). Interestingly, the concentrations of Cu^2+^ delivered from microparticles were higher than Co^2+^. As expected, maintaining the low concentration of Cu^2+^ and increasing the concentration of Co^2+^ (low Cu (MP)-high Co (F)), the concentration of Ca^2+^ was reduced whereas the concentrations and release profile of Cu^2+^ were maintained (Figure 7C). Curiously, the release of Co^2+^ from the fiber presented zero-order kinetics, with higher concentrations delivered compared to Cu^2+^ at the end of the experiment. Unexpectedly, with high Cu^2+^ concentrations within the microparticles and low Co^2+^ within the fiber (high Cu (MP)-low Co (F)), the amount of Cu^2+^ released was higher than Ca^2+^ (Figure 7D). Moreover, the amount of Co^2+^ released was lower compared to the previous condition where the same amount of Co^2+^ was incorporated (low Cu (MP)-low Co (F)) (Figure 7B,D). Finally, maintaining the high concentration of Cu^2+^ within the microparticles and incorporating high concentration of Co^2+^ within the fiber (high Cu (MP)-high Co (F)), the amount of Cu^2+^ released was maintained the same (Figure 7D,E). Additionally, the amount of Ca^2+^ was reduced compared to Cu^2+^ and all previous conditions tested. Interestingly, the release profile of Co^2+^ presented zero-order kinetics (Figure 7E). However, the amounts released where lower than a previous condition where the same amount was incorporated (low Cu (MP)-high Co (F)) (Figure 7C), although in that case, the amount of Cu^2+^ incorporated was lower.

## 4. Discussion

In this study, we aimed to develop a multiple ion delivery system platform to be used to allow the delivery of several cues from scaffold-based biomaterials. For this purpose, we first aimed to develop microparticles with an osteogenic composition that could potentially be used for bone regeneration purposes and incorporate them within alginate. As a proof of concept, we introduced three therapeutic ions, used as model ions, within the ion delivery system to study if the material design was appropriate to control and deliver those ions. More specifically, the ions introduced were Cu^2+^ and Co^2+^, which could eventually promote antibacterial and angiogenesis response, respectively. Moreover, Ca^2+^ was also incorporated as being reported to be angiogenic and osteogenic.

Initially, we aimed to develop microsphere-like particles using a method similar to spraying method to eject the liquid microparticle composition as droplets. In this sense, we used a fingertip spray bottle to eject the alginate/HA blend into a divalent ion crosslinking solution to instantly gel the droplets. To the best of our knowledge, there is only one study reporting the use of a fingertip spray bottle as well, generating pure alginate microspheres containing cells [24]. Our results demonstrated practically the same size distribution between triplicates with all alginate/HA blends tested, with a low coefficient of variation of the mean size (Figure 2), indicating high reproducibility of this method. However, with this approach we obtained a broad distribution size of microparticles compared to other sophisticated methods, such as electrospraying or microfluidics, in which a narrow size distribution is obtained [25,26]. Nonetheless, applying sieving in a narrow range after their development can nearly overcome this. Additionally, the results of how HA influences the mean size of microparticles (Figure 2) is similar to another study, where it was also found a decrease size of alginate/HA beads when the amount of HA was increased [27].

As HA has been described to be osteoconductive and enhance osteogenic differentiation and bone formation with MSC [28,29,30], we aimed to introduce the maximum HA amount to improve the osteogenic properties of the microparticles and mimic the native inorganic composition of bone. Some studies developed microparticles with 100% HA composition; however, it involved the use of hydrothermal processing or sintering [31,32], which increases the amount of smooth surface and reduces the ability to degrade and release possible ions incorporated as well as limits the incorporation of molecules within the structure due to water soluble toxic binders used and high temperatures. A way to overcome this limitation is to develop microcarriers at room temperature with non-toxic binders, which at the same time have interconnected pore structure. Similar to our approach, other studies developed alginate/HA microspheres with lower ratios, specifically 1:15 [33] or 95:5 and 50:50 [27]. Our microparticle composition of choice had an alginate:HA ratio of 1:40, which is higher than the previous studies mentioned. In our case, our aim was to incorporate the maximum HA amount to potentially stimulate osteogenesis and, at the same time, use the minimum amount of alginate as a binder containing the desired amount of ions. It is well known that the combination of HA sources is fundamental for successful bone regeneration, generally using autologous and allogenic bone [34], although their limited availability urge for the use of synthetic sources such as the present scaffold.

**Figure 8:**
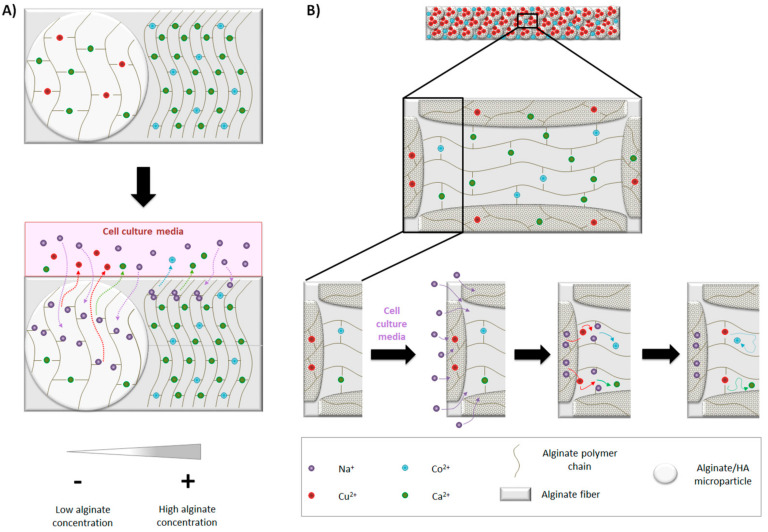
Ion release. (**A**) Effect of cell culture media on ion delivery: Na^+^ is able to diffuse easily into microparticles compared to alginate fiber due to lower alginate concentration. Na^+^ gradually replaces divalent ions, resulting in the un-crosslinking of alginate polymer chains, facilitating the release of divalent ions. (**B**) Influence of Cu^2+^ on Co^2+^ and Ca^2+^ release: in the presence of cell culture media, Na^+^ replace divalent ions from microparticles and alginate fiber. The delivery of Cu^2+^ from microparticles crosslinks free alginate chains from alginate fiber due to its higher affinity. Ca^2+^ and Co^2+^ remain entrapped and have more difficulties to diffuse.

As a final step, we aimed to assemble the multiple an ion delivery platform by incorporating 1:40 microparticles containing different amounts of Cu^2+^ within alginate fibers containing different amounts of Co^2+^. Their pattern release was studied together with Ca^2+^, also present either within microparticles and alginate fiber. In general, Ca^2+^ and Cu^2+^ presented a burst release, whereas Co^2+^ presented zero-order kinetics release. Additionally, higher amounts of Cu^2+^ were released compared with Co^2+^ when same concentrations were incorporated in their respective crosslinking solutions (Figure 7B,E), which can be due to different reasons. One of them is that the final concentration of alginate within microparticles was 0.5%, whereas in the fiber was 1.5%. It is described that stability of alginate can be decreased when in contact with monovalent ions such as Na^+^, as they can replace Ca^2+^ and unbind alginate chains [35]. Since we performed the release study with cell culture media and it contains a concentration of NaCl approximately of 150 mM, alginate from microparticles (0.5%) could be degraded faster than the alginate fiber (1.5%) and release more Cu^2+^ (schematic representation in Figure 8A). Furthermore, as alginate fibers are fully loaded with microparticles (50%*v*/*v*) and most of them reach to the fiber surface, there might be a direct delivery of Cu^2+^ ions to the medium.

Another observation is that Cu^2+^ seems to have an influence in Co^2+^ and Ca^2+^ release. In more detail, with the same amount incorporated of Co^2+^, when the amount of Cu^2+^ is increased, the quantity of Co^2+^ was reduced (Figure 7BE). Moreover, for the same concentration of Cu^2+^ and Co^2+^ introduced in the crosslinking solutions, Cu^2+^ seemed to reduce more the delivery of Ca^2+^ (Figure 7). Conversely, Co^2+^ seemed to not interfere with Cu^2+^ release, as the delivery pattern was practically the same with the same amount of Cu^2+^, independently of the Co^2+^ amount incorporated in the fibers (Figure 7B–E). The rationale behind the decreased release of Co^2+^ and Ca^2+^ in the presence of Cu^2+ +^ is not completely understood. However, we speculated that it could be due to ion affinity to alginate. It is described that the gel forming ability follow this order: Cu^2+^ > Ca^2+^ > Co^2+^ [36,37]. Therefore, we hypothesized that the delivery of Cu^2+^ from inner microparticles might be further crosslinking alginate chains, probably reducing the mesh size, which is described to lower the diffusion rate of the encapsulated molecules, in our case Ca^2+^ or Co^2+^ [38,39] (schematic representation shown in Figure 8B). Interestingly, the order kinetics of Co^2+^ release was close to zero. Probably, due to the high amount of alginate/HA microparticles incorporated within alginate fiber and the higher affinity of Cu^2+^ for alginate, Co^2+^ might have had more difficulties to diffuse and, therefore, it presented a more controlled and sustained release. These results suggest that the type of divalent ion used with the crosslinking of alginate-based scaffolds might influence the release of other ions and it should be taken into account.

The current work pretended to design a platform for the delivery of multiple ions, although will also be useful for the multiple delivery of molecules. As it was designed as a delivery platform, the final application can vary depending on the regeneration steps that can be tackled. Our previous finding performed with similar studies have shown that the released doses of ions are in non-cytotoxic levels. Furthermore, we understand that, depending on the applications, the released doses may be found in therapeutic ranges, which will allow triggering the specific desired processes. In our ion delivery platform, we used Cu^2+^ to potentially provide anti-bacterial properties [12], Co^2+^ to potentially stimulate blood vessel formation [15] and Ca^2+^ to potentially stimulate angiogenesis and osteogenesis [7,40,41]. Therefore, the ion delivery system developed in this study has the potential to be used as a bioactive ion delivery system for bone regeneration. Regarding the concentrations released, several Cu^2+^ concentrations are reported to be effective against different bacterial strains, from 150 to 6000 µM, approximately [42,43,44]. This would correspond to the condition where 1 mM of Cu^2+^ (low Cu^2+^ (MP)) was incorporated in the crosslinking solution (Figure 7B,C), where practically all Cu^2+^ (≈500 µM) was delivered within 24 h. Regarding Co^2+^, concentrations from 10 to 100 µM are reported to stimulate the angiogenic response when delivered from scaffolds [18,45]. Considering our results, the condition in which 1 mM of Cu^2+^ and Co^2+^ (low Cu^2+^ (MP)—low Co^2+^ (F)) were incorporated in the cross-linking solutions resulted in a zero order kinetics release of Co^2+^ during 7 days with constant concentrations of approximately 30–40 µM (Figure 7B). Therefore, this condition has the potential to stimulate the angiogenic response. Finally, considering Ca^2+^, the maximum amount delivered was around 5 mM (Figure 7A), which is higher than the concentration found in blood plasma or extracellular fluids (around 2 mM). However, a previous study reported that whereas concentrations of 2–4 mM were suitable for osteoblast proliferation, concentrations from 6–8 mM enhanced osteoblast differentiation and matrix mineralization [46]. Our system seems to be promising to stimulate bone formation when only Ca^2+^ or Ca^2+^ with the lowest concentrations of Cu^2+^ and Co^2+^ (1 mM) are incorporated in the crosslinking solutions.

## 5. Conclusions

To summarize, we have been able to develop a platform containing HA microparticles, being a highly reproducible approach. Moreover, when incorporated within alginate fibers, and different ions were added within microparticles and alginate fibers allowing multiple delivery of therapeutic ions, tuning the ion release through their concentrations in the crosslinking solution. The designed platform can be further tuned to the desired ion release varying the different parameters such as crosslinking solution, crosslinking time, alginate concentration or fiber size among others. This tuning, allowing the controlled release of different therapeutic ions at different doses, will eventually allow modulating different steps of tissue regeneration processes in tissue engineering.

## Figures and Tables

**Table 1:** Different combinations of Ca^2+^, Cu^2+^ and Co^2+^ in crosslinking solutions of alginate/HA microparticles and alginate fibers. Abbreviations: MP (microparticles); F (fiber).

Sample Code	Microparticle Crosslinking Solution		Fiber Crosslinking Solution	
	CaCl_2_ (mM)	CuCl_2_ (mM)	CaCl_2_ (mM)	CoCl_2_ (mM)
Control Cu (MP)—Control Co (F)	150	0	150	0
Low Cu (MP)—Low Co (F)	149	1	149	1
Low Cu (MP)—High Co (F)	149	1	140	10
High Cu (MP)—Low Co (F)	140	10	149	1
High Cu (MP)—High Co (F)	140	10	140	10

## Data Availability

Data is contained within the article or supplementary material.

## Supplementary Materials

The following are available online at https://www.mdpi.com/article/10.3390/ma14247676/s1, Table S1: Crosslinking solution composition for Alginate/HA microparticle formation, Table S2: Crosslinking solution composition for alginate fiber formation.
