# Supplementary material for: Multiple Ion Scaffold-Based Delivery Platform for Potential Application in Early Stages of Bone Regeneration
Source: Materials (Basel). 2021 Dec 13;14(24):7676. doi: 10.3390/ma14247676 (PMC8706177; doi:10.3390/ma14247676)
Supplement: Supplementary file 1 [file materials-14-07676-s001.zip › materials-1425788-supplementary.pdf]

Supplementary Table S1. Crosslinking solution composition for alginate/HA microparticle formation. Concentration of divalent cations of Ca^{2+} and Cu^{2+} was varied to crosslink alginate with HA and incorporate Cu^{2+}. Abbreviations: MP (microparticles).

Sample code	$CaCl_2$ (mM)	$CuCl_2$ (mM)
**Control Cu (MP)**	150	0
**Low Cu (MP)**	149	1
**High Cu (MP)**	140	10

Supplementary Table S2. Crosslinking solution composition for alginate fiber formation. Concentration of divalent cations of Ca^{2+} and Co^{2+} was varied to crosslink alginate and incorporate Co^{2+}. Abbreviations: F (fiber).

Sample code	$CaCl_2$ (mM)	$CoCl_2$ (mM)
**Control Co (F)**	150	0
**Low Co (F)**	149	1
**High Co (F)**	140	10
